# Supplementary material for: Antiviral kinetics of tenofovir alafenamide and tenofovir disoproxil fumarate over 24 weeks in women of childbearing potential with chronic HBV
Source: PLoS One. 2021 May 13;16(5):e0251552. doi: 10.1371/journal.pone.0251552 (PMC8118264; doi:10.1371/journal.pone.0251552)

**S1 Table. Univariate analysis.**

**Predictors of HBV DNA ≥ 200,000 IU/mL at Week 12**


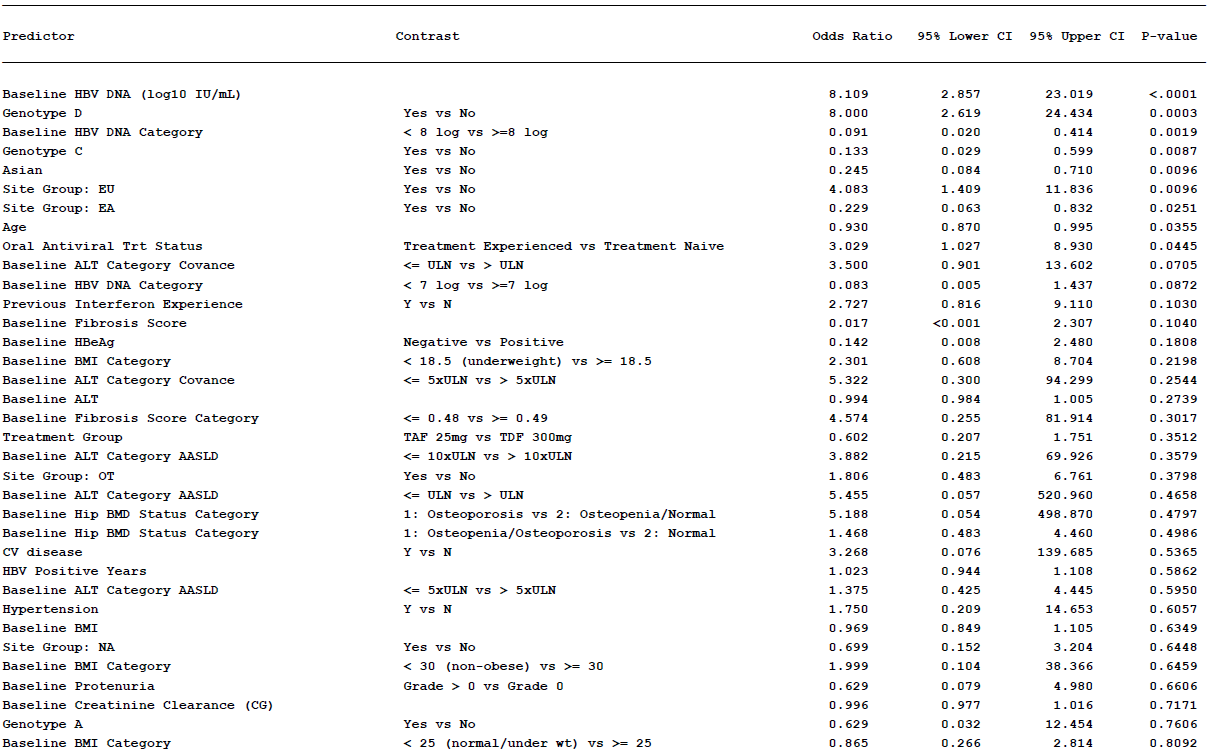


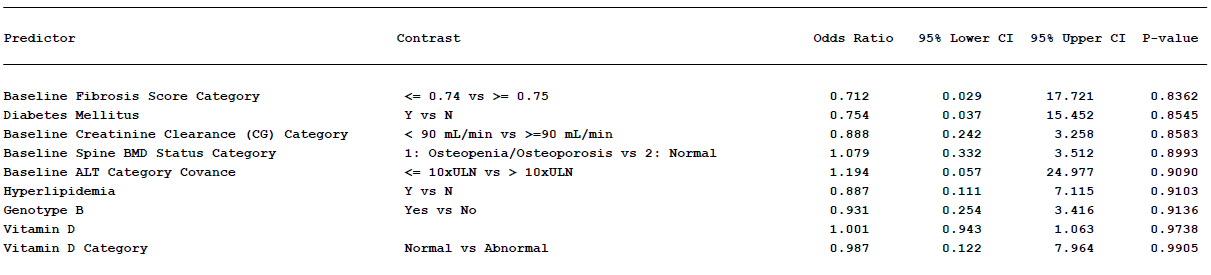


**S1 Table. Univariate analysis (continued)**

**Predictors of HBV DNA ≥ 200,000 IU/mL at Week 24**


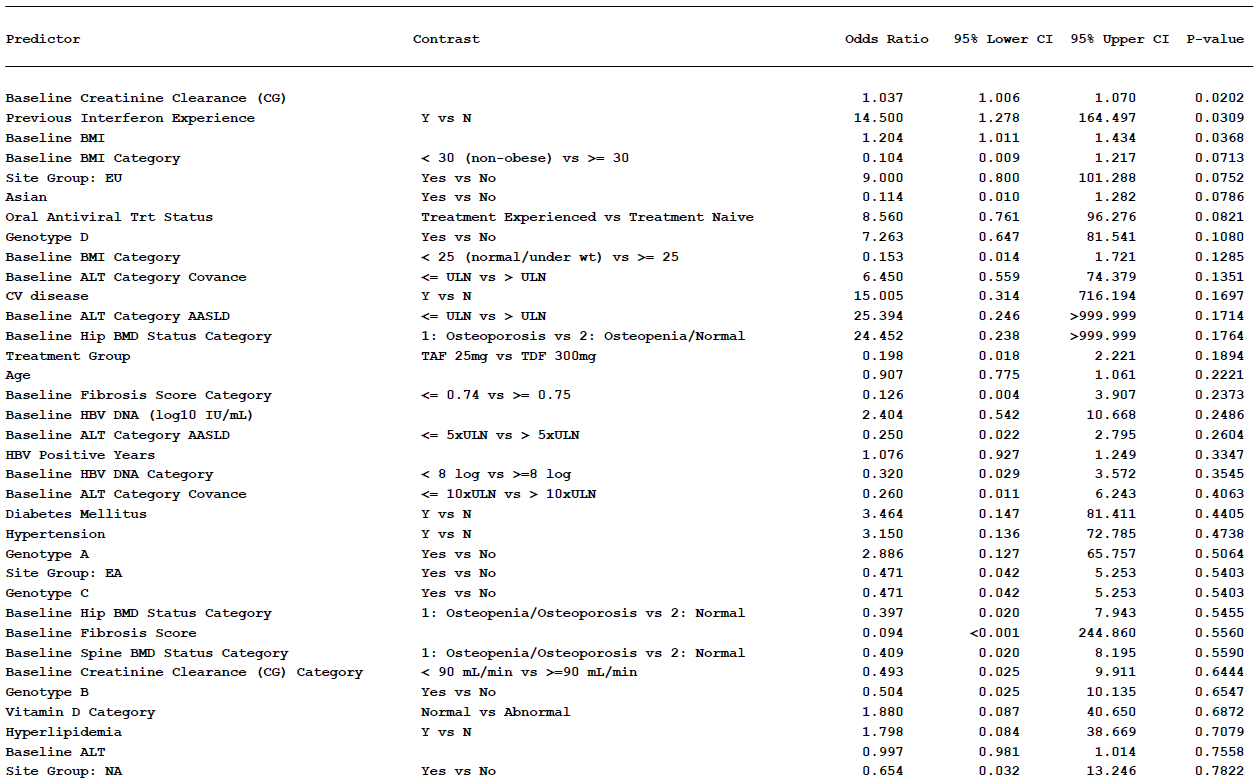


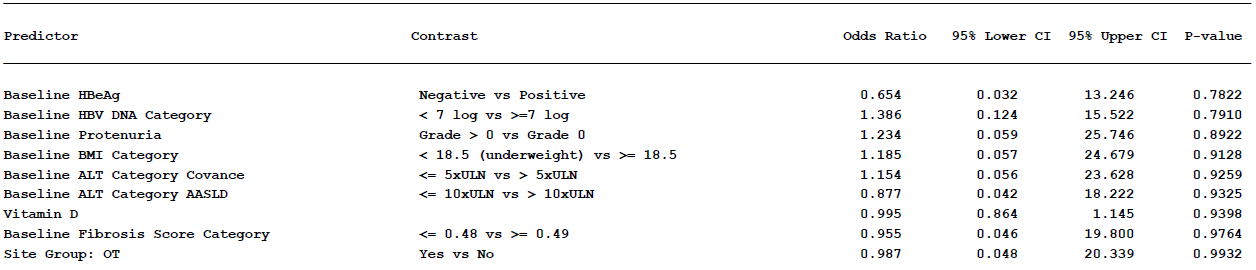

Supplement: S1 Table — Predictors of HBV DNA ≥ 200,000 IU/mL at Week 12. (DOCX) [file pone.0251552.s001.docx]
