# Supplementary material for: Antiviral kinetics of tenofovir alafenamide and tenofovir disoproxil fumarate over 24 weeks in women of childbearing potential with chronic HBV
Source: PLoS One. 2021 May 13;16(5):e0251552. doi: 10.1371/journal.pone.0251552 (PMC8118264; doi:10.1371/journal.pone.0251552)
Supplement: S1 Fig — SD, standard deviation; TAF, tenofovir alafenamide; TDF, tenofovir disoproxil fumarate. *p<0.05, †p<0.001. (DOCX) [file pone.0251552.s002.docx]

**S1 Fig. Changes from Baseline in Estimated Glomerular Filtration Rate (eGFR) by the Cockcroft-Gault Method.**

SD, standard deviation; TAF, tenofovir alafenamide; TDF, tenofovir disoproxil fumarate. *p<0.05, †p<0.001.
